# Mental Health Support in the Time of Crisis: Are We Prepared? Experiences With the COVID-19 Counselling Programme in Hungary

**DOI:** 10.3389/fpsyt.2021.655211

**Published:** 2021-05-31

**Authors:** Zsófia Szlamka, Márta Kiss, Sámuel Bernáth, Péter Kámán, Amina Lubani, Orsolya Karner, Zsolt Demetrovics

**Affiliations:** ^1^Institute of Psychology, ELTE Eötvös Loránd University, Budapest, Hungary; ^2^Department of Psychology, Institute of Psychiatry, Psychology and Neuroscience, King's College London, London, United Kingdom; ^3^Counselling Centre, ELTE Eötvös Loránd University, Budapest, Hungary; ^4^Centre of Excellence in Responsible Gaming, University of Gibraltar, Gibraltar, Gibraltar

**Keywords:** counselling, COVID-19, crisis mental health, online counselling, brief intervention

## Abstract

The Coronavirus disease 2019 (COVID-19) posed unexpected global economic and societal challenges. These include a heavy impact on mental health due to fast changing lockdown and quarantine measures, uncertainty about health and safety and the prospect of new waves of infections. To provide crisis mental health support during the pandemic, Eötvös Loránd University in Hungary launched a specialist online counselling programme, consisting of one to three sessions. The programme was available to all university members between 4th March and 25th May 2020. Overall, 47 clients received support. In this paper we discuss challenges reported by clients, key features of providing a brief mental health intervention online, reflect on counsellor experiences and give recommendations on how mental health services could be developed in the time of crisis. Most clients had challenges with developing a daily routine under quarantine; and many had hardship related to finances, housing, and distance learning. Common mental health consequences included fear from the virus and stress, anxiety, and fatigue due to the interruption to everyday life. In some cases, more complex conditions were triggered by the pandemic. Examples include addictive behaviours and symptoms of depression or psychosis. However, referring cases beyond the competency of counselling proved to be a challenge due to the closure of specialist services. Counsellors observed three key features to the online delivery of a brief crisis mental health intervention: [1] an explicit problem-oriented approach to counselling; [2] challenges of building rapport online; and [3] frames of online counselling. Counsellor experiences often overlapped with those of clients and included challenges of working from home and adjusting to online counselling methods. The possibility of online counselling allowed that mental health care could take place at all during the pandemic. Client experiences reflect findings from previous literature. Like other mental health initiatives launched to tackle COVID-19, the intervention's effectiveness was not measured given the unexpected context and short time frame for programme development. We recommend the use of impact measurement tools to develop mental health services in crises. Meanwhile, the pandemic brought to attention the need to better understand online delivery models. Counsellors should have access to training opportunities on online counselling and managing work-life balance in a remote setting. The COVID-19 counselling programme in Eötvös Loránd University, Hungary is an example of providing online mental health counselling in the time of crisis. Clearly, more studies are needed discussing delivery models and effectiveness of mental health interventions during the pandemic. Experience and knowledge sharing across practitioners should be encouraged to improve how the field reacts to unexpected, high risk events and crises.

## Introduction

The Coronavirus disease 2019 (COVID-19) posed unexpected challenges globally. Communities live under fast changing lockdown and quarantine measures, uncertainty about health, and a prospect of a second wave of infections. COVID-19 is a low probability, unprecedented event with immense consequences on mental health (1). While providing mental health care for all has long been promoted by the global mental health community (2), the crisis brought attention to the immense need of developing and improving mental health services worldwide. Researchers and practitioners pointed out the timeliness of developing sustainable mental health care delivery systems (3, 4), and asked for a stronger emphasis on mental health research (5). The United Nations also contributed to the discussion about improving mental health services with the increased needs of the pandemic and called for a strategic shift regarding the historic underinvestment in mental health (6).

Many of the mental health-related consequences of COVID-19 have been reported globally, across a range of cultures and contexts. These include for example increasing levels of mental distress and anxiety due to the uncertainty (3, 7, 8), depressive symptoms, poor sleep quality (9), and a fear of the virus (10, 11). The psychosocial impact of the crisis include health anxiety due to COVID-19-related media content and exposure to social media (12, 13); burnout among medical staff and health workers (14–16); and fear of health consequences among COVID-19 patients (17). It is suggested that the pandemic may act as a trigger to symptoms of pre-existing mental health conditions (18) and to suicidality (19). Problematic internet use has also become increasingly common (20, 21). The pandemic has brought to light existing health inequalities across contexts (22). Groups that are particularly impacted include for example people infected and their families; medical staff; people living with disabilities or chronic psychiatric conditions; those at a low socio-economic level (23); children and adolescents (24); and those coming from ethnic minority groups (22, 25).

There are some regional variations reported regarding the extent to which the mental health of communities is impacted. As an example, the level of anxiety that health care workers in COVID-19 health departments experience differs in an Asian as compared to a Central European context (26, 27). Further, there is a varying amount of evidence available in English for a global audience from different geographies. For example, while there is some limited information available about the mental health consequences of COVID-19 in Central and Eastern Europe (28–30), studies on pandemic-related mental health interventions are lacking.

Spatial distancing worldwide means a turning point in providing mental health care: services are delivered using online methods, such as apps or eHealth platforms (1). E-health-based tools are proven to be effective in addressing misinformation and reaching audiences across socio-economic contexts (31). However, mental health professionals who were not previously familiar to eHealth had to suddenly learn and use remote delivery methods (32, 33). Researchers emphasise the need that public health focuses on eHealth literacy among communities for better service uptake (34). Services launched during the pandemic include for example online mental health education on coping with distress (35), self-help programmes using social media and online counselling (36, 37). However, little is known about how these educational, self-help, and counselling programmes were developed and what short-or long-term impact they had on beneficiaries.

To tackle the mental health-related consequences of the pandemic, Eötvös Loránd University (ELTE) in Hungary launched its COVID-19 counselling programme as part of its Counselling Centre (CC). The aim of this specialist service was to offer immediate and problem-focused support with coping with the crisis. In this paper our goal is to share experiences and reflect on the COVID-19 counselling programme based on observations of our counsellors. We aim to offer recommendations on how services responding to mental health crises can be developed in a timely and efficient manner, addressing the needs of beneficiaries.

## Context

Eötvös Loránd University's COVID-19 counselling programme was made available to all university members, including students, employees, and members of partner organisations. This specialist service consisted of one to three online sessions in Hungarian or in English; it ran between 04.03.2020 and 25.05.2020; and was funded by ELTE. The counselling frames were designed based on the counselling support that the CC normally offers to all university members: including up to six sessions and taking an eclectic approach to counselling (38) (for a detailed description and comparison with the COVID-19 counselling programme, see Table 1). The COVID-19 counselling programme kept this eclectic nature and included person-centred, psychodynamic, cognitive-behavioural, and crisis interventional elements. Counselling was provided primarily by members of the CC (two counselling psychologists and two clinical psychologists). Seven other psychologists affiliated to ELTE joined the efforts on a voluntary or contractual basis to cater for the need triggered by COVID-19. The programme was operating in parallel with the usual six-session-long service.

**Table 1:** Comparing the COVID-19 counselling programme with the six-session-long counselling service provided by CC.

	**Six-session-long counselling service**	**COVID-19 counselling programme**
Number of sessions	1–6 sessions, typically 6	1–3 sessions
Interface	In person or online	Online
Focus of counselling	Focus is developed jointly by client and counsellor	Explicit: coping with the pandemic
Key features	Eclectic	Eclectic
The counselling process	First interview Exploration and a situational diagnosis Elaboration of the focus and contract Working phase with counselling interventions Closure	First session: exploration of the problem and support the client to express feelings 2nd and 3d sessions: devoted to the elaboration of personal coping strategies and best practises

University members could register to the CC online. Upon registration, clients could choose from receiving immediate support related to coping with the pandemic or the general, six-session-long counselling programme. Clients were offered support on a first come first served basis. Overall, 47 clients received support (for details see Table 2). The CC ended the specialist service upon the ease of the lockdown after the first wave of infections as at that point no further lockdowns were expected.

**Table 2:** Participants of the COVID-19 counselling programme.

**Beneficiaries**	**Applied to the programme**	**Attended the programme**
Local students	39	35 (31 female, 4 male)
		16 Bachelor students, 18 Master, 1 PhD students
International students	12	11 (8 female, 3 male)
		5 Bachelor students, 4 Master, 2 other students
Employees	1	1 (1 female)
Total	52	47 (40 female, 7 male)

Beside the one-on-one counselling support, the CC developed a psychoeducational guide aiming to improve resilience and well-being among university members by offering a range of coping strategies (39). This guide was sent to all university members by emails and it was made available on the university's website and Facebook pages.

In June 2020, upon completion of the service, all counsellors involved in the COVID-19 counselling programme were consulted and asked to share their experiences with taking part in a such a brief, online service. The consultation took place online and key experiences and observations were summarised in the form of written notes. The authors analysed counsellor experiences by looking for key patterns and these patterns were developed in iterations among authors.

## Experiences With the Covid-19 Counselling Programme

Counsellors of the COVID-19 counselling programme observed three key patterns: (a) challenges reported by clients; (b) features of an online, brief counselling programme; (c) experiences of a counsellor in the time of crisis. Table 3 provides a summary of counsellor observations and the next section will discuss these features in detail.

**Table 3:** Summary of counsellor observations.

**Challenges reported by clients**	**Interruption to everyday life**	
	Mental health-related consequences	Complex issues uncovered
Features of an online, brief counselling programme	Focus and elements of counselling	
	Building rapport	
Experiences of a counsellor in the time of crisis	The frames of online counselling	

### Challenges Reported by Clients

Most of the clients reported challenges in the following areas: (a) interruption to everyday life; and (b) mental health-related consequences.

#### Interruption to Everyday Life

An overarching concern was the major interruptions to everyday life. Many found it difficult to develop a new daily routine in quarantine, experienced financial and housing-related hardships, or troubles with distance learning.

Most clients found it challenging to adjust to a daily routine in quarantine: structuring the day and finding the balance between productivity and recreational activities at home proved to be difficult. Many reported conflicts with family members or partners. Others experienced changes in eating and sleeping habits: how many times a day they ate, at what time they went to sleep or woke up. Certain clients, particularly extraverted students, experienced mental health difficulties due to the separation from their community and the absence of social activities. Others found that the fact that all social gatherings were cancelled relieved stress since they did not fear missing out.

Most clients reported financial or accommodation-related challenges. Many students who used to work beside their studies lost their job and found it difficult to make ends meet. This hit international students from lower socio-economic backgrounds especially hard. Due to the closure of dormitories, many had to leave their accommodation until further notice and move back with their family. This uncertainty about housing and the lack information about when students could return caused trouble for many. International students, often residing in Hungary on a limited residence permit, felt anxious about constantly changing travel restrictions while being in quarantine in a foreign country. Meanwhile, Hungarian students on exchange programmes were stressed because of being in quarantine abroad, often in highly infected areas.

Finally, some clients had challenges with distance learning. They experienced an increased workload while not having clear channels of communication with other students and teachers. Many felt that they lacked the self-discipline needed to keep up with their work. Those who finished their studies were unsure about how final exams would take place and their future career opportunities also remained uncertain.

#### Mental Health-Related Consequences

While most clients came across at least one of the difficulties described above, their lived experience differed greatly. Overall, counsellors found it difficult to decide what behaviours could be considered as a normal human reaction to crisis and when symptoms reached clinical severity. Clients' mental health was impacted by the presence of the virus, rapid changes, and uncertainty as to how long the pandemic will last and what impact it will have. Fear, anxiety, and even panic attacks were reported frequently. Other problems included depressive mood, fatigue, eating and sleeping disturbance, hopelessness, loss of interest and motivation, and occasionally impulsive behaviour. Group dynamics only made symptoms worse: for example, students in dormitories reported an elevated sense of fear and stress due to constant group discussions about COVID-19.

#### Complex Issues Uncovered

For a few clients, the pandemic acted as a trigger to pre-existing mental health conditions. In these cases, the counselling process rapidly uncovered more complex problems behind symptoms experienced during quarantine. For example, counsellors observed an increase in psychotic symptoms, the use of psychoactive substances, and porn addiction. The three sessions offered by the COVID-19 counselling programme were not enough to manage these complexities and therefore these clients were referred to specialist services. However, due to the closure of many public and private health departments it was nearly impossible to find available specialist care.

Overall, most of the clients found it difficult to adjust to life in quarantine and develop a new daily routine. Mental health-related consequences included symptoms such as fear, stress, fatigue, and depressive mood. On occasion more complex problems were uncovered and referred to specialist care. Referral was difficult because of the closure of many health departments due to COVID-19.

### Features of an Online, Brief Counselling Programme

The COVID-19 counselling programme had many unique characteristics as compared to the longer, more in-depth service that the CC normally offers. On the one hand, it was significantly shorter with only one to three sessions, it took place virtually and it had a pandemic-oriented focus. The brevity and the online nature of the programme led to novelties in counselling such as: (a) changes in the focus and elements of counselling; (b) new ways of building rapport; and (c) changes in the frames of online counselling.

#### Focus and Elements of Counselling

Firstly, the programme had an explicit focus on coping with the crisis. This is different from a service in which the focus is developed together by the client and the counsellor as part of the counselling process. We used a crisis interventional approach to counselling. On the one hand, this allowed counsellors to create a space for clients in which they felt comfortable expressing emotional reactions regarding the ongoing lockdown. On the other hand, this approach also allowed for bringing cognitive, psychoeducational perspectives into counselling so that clients can find personality-oriented coping strategies.

The brevity of the programme resulted in a more coaching-like intervention with an accelerated counselling process. The first session allowed for the exploration of the consequences of the pandemic, while the counsellor offered emotional support. The second and third sessions had an emphasis on personal coping strategies and best practises to maintain well-being during a crisis. Meanwhile, the time limitation did not allow for a deeper and less directive exploration of difficulties or more the use of complex interventions. Given the novel delivery method of the intervention, counsellors expressed the importance of supervision in between sessions.

#### Building Rapport

Building rapport was challenging: many counsellors thought that the brevity and the online environment of the programme led to a distant client-counsellor relationship, especially when comparing to the six-session-long service. For example, there was no time for a complete first interview and the high turnover of clients meant that it was challenging to develop a deeper relationship with clients. Some counsellors felt pressured to achieve visible progress over the course of only three sessions and this may have impacted how they built rapport.

The online delivery of counselling had a unique impact on building rapport. In most cases the video chat allowed clients and counsellors to see one another's face. However, metacommunication (such as gestures and body movements) and contextual clues (such as clothing) were oftentimes missing from online communication. Some felt that this restricted contact negatively impacted rapport or made the relationship feel as unrealistic. At times, weak internet connexion distorted the video chat, or the connexion was interrupted, influencing rapport and the effectiveness of counselling.

The online environment also brought about some advantages. It offered a different way of building rapport: instead of meeting in the counsellor's office, the venue of the counselling was one another's home or private space. Many clients thought that this allowed for more intimacy, although it made others feel uncomfortable.

#### The Frames of Online Counselling

The brief, online delivery model brought about changes in the frames of counselling [the fixed elements, the boundaries and the context of the counsellor-client relationship set out for the client's benefit (40)]. The online aspect meant that it was harder for the counsellor to control the frames. Counselling often had to take place in unusual settings so that both the client and the counsellor can be alone. Examples include in a park, on the street, in a car or even in the bathroom. Family members or roommates may have interrupted the session, or the client did not feel comfortable speaking freely due to the presence of others next door. Some clients experienced fatigue due to the heavy online presence during the day and many reported a difficulty of shifting focus from work to counselling.

For others, attending sessions from their private space made them feel more relaxed and this facilitated self-reflection. Clients with social anxiety added that it was easier for them to engage in counselling and talk freely on an online platform. Meanwhile, the online model meant less cancellations and clients arrived more on time compared to in-person counselling.

In sum, the COVID-19 counselling programme differed from in-person counselling in its brevity and online nature. Changes included an explicit focus on coping with the crisis, a difficulty in building rapport with clients, and new frames in counselling.

### Experiences of Counsellors in the Time of Crisis

Conditions imposed by the pandemic affected counsellors as well: they had to manage the impact of the crisis on their personal life, adapt to remote work and face an increased risk for burnout.

Counsellors were exposed to the same crisis that they were supposed to help manage and were left with little time to cope with difficulties in their own life. Many of the challenges they experienced overlapped with those of clients. Some colleagues found it difficult to structure their day and separate work and recreational activities. For others, the presence of family or partner made it hard to talk freely. At times, working remotely meant disturbing noises during counselling, such as renovation from the background. Many counsellors found that it was a novel professional challenge to let clients virtually enter their home. However, certain colleagues found it easier and more comfortable to prepare for sessions remotely. In general, the blurred boundaries between work and private life, sitting in front of the computer the whole day and the lack of social and recreational activities challenged counsellors' own mental health.

The similarity of experiences with clients led to a risk of distraction by the counsellors' own feelings or becoming too involved with the client's individual circumstances, hence losing the emotional distance necessary to help. Moreover, counsellors lacked previous training regarding crisis interventions and the mental health consequences of a public health crisis.

The rapidly changing working conditions and the high turnover of clients increased the counsellors' risk of burnout. Some counsellors experienced pressure to achieve quick and visible progress in a short timeframe. Others felt that the programme's brevity hindered getting more deeply involved with the counselling process. Many thought that the heavy focus on the pandemic made the process become somewhat repetitive. These difficul ties challenged the motivation of counsellors: most of them reported increased fatigue. Besides, many counsellors experienced loneliness, missed the in person contact with clients and the support of colleagues with counselling.

## Discussion

Despite the fact that there have been severe mental health consequences of the COVID-19 pandemic, there is a lack of empirical evidence about the features of providing mental health counselling in the time of crisis. The COVID-19 counselling programme provided by ELTE in Hungary offered mental health support to university students and employees with a focus on coping with the pandemic. In this paper we discussed three key features of the service: challenges reported by clients, features of a brief, online counselling programme, and experiences of counsellors during the pandemic.

The majority of clients had difficulties with developing a daily routine in quarantine and many had challenges with making ends meet, housing, or distance learning. Due to the rapid changes and uncertainty of how the crisis evolves, clients' mental health was severely affected. Symptoms reported the most frequently included fear, stress, and anxiety. These are symptoms that similar studies from across the globe have been widely reporting (11, 12, 37). Other experiences included depressive mood, fatigue, disturbed eating and sleeping habits and a loss of motivation and interest, in line with previous work (7, 8, 37). At times, pre-existent mental health problems were revealed by counselling, such as psychotic symptoms, addictive behaviours, and problematic internet use or substance abuse. Such cases were also reported in studies investigating mental health care during COVID-19 (18).

The vast majority of clients taking part in our programme were students: most of them are not carers and are financially dependent on their family. Therefore, caring responsibilities and financial problems were much less represented in their views compared to the general population (41, 42). On the other hand, students were generally worried about the lack of social activities and their future career opportunities, especially those closer to graduation. International students would have benefitted from special care being in quarantine in a foreign country and being even more prone to financial- and housing-related uncertainty.

Due to its brief and online nature, counselling had a set of unique features as compared to in-person care. First, its explicit focus was to help clients cope with the pandemic. Counsellors took a cognitive, problem-oriented approach with elements of psychoeducation, coaching, and crisis intervention. They also aimed to help clients relieve distress and express emotions, while building personality-oriented coping strategies. Building rapport was challenging in an online environment: metacommunication and contextual cues were regularly missing from communication. The frames of counselling also changed due to the online environment and counsellors had less control over them. Sessions often had to take place in unusual settings so that clients and counsellors can talk freely and comfortably.

Finally, counsellors' experiences often overlapped with those of clients: they had to adjust to remote work and address the impact of crisis on their personal life. This overlap put them at a risk of losing the necessary distance from clients so that they can best support them in counselling. Counsellors were also at a risk of burnout and fatigue due to the high turnover of clients, a lack of training on addressing a crisis, and blurred boundaries between work, private life, and limited recreational possibilities. Burnout has been extensively discussed in literature regarding medical staff (43).

## Implications

The COVID-19 counselling programme's development was a rapid reaction to the quickly evolving pandemic. The possibility of running counselling online allowed that mental health care could take place at all given the conditions. As the programme only consisted of 3 sessions, a larger number of people in need could be reached. It also allowed for easy access to mental health care and short waiting times for beneficiaries. A summary of the key implications of this work can be found in Table 4.

**Table 4:** Key implications.

**Key implications**
Mental health interventions should be evaluated for effectiveness even in the time of crisis
More research is needed to understand the implications of remote counselling services
Counsellors should be trained on providing support remotely and respond to a societal crisis with mental health consequences.
There is a need for socially distanced specialist psychiatric services in the time of crisis.

The question remains as to how counselling can better react to unprecedented, high risk events, and crises. Literature recently published about mental health services during the pandemic suggest that psychological interventions are not adequately planned and coordinated in a crisis like COVID-19; and there is a lack of focus on intervention implementation and professionals trained in crisis management (44). Our experience with the COVID-19 counselling programme is aligned with this. ELTE's Counselling Centre developed the intervention with the intention of offering support as quickly as possible, without a strong emphasis on implementation frameworks. Overall, clients reported that they found the COVID-19 counselling programme beneficial despite all the challenges. There is an abundance of published work drawing attention to the need to invest in mental health services and a growing literature evaluating intervention effectiveness in COVID-19 (45). However, there is not enough quantitative evidence about the effectiveness of brief online interventions in crisis management. Likewise, there are only few reports on practical experiences with mental health counselling during the crisis. We therefore encourage experience and knowledge sharing across practitioners. We also suggest the use of impact measurement tools in the field of mental health counselling in any next crisis or potential second wave.

The pandemic brought attention to the need of better understanding online services. Online counselling proved to be helpful for our clients with social anxiety. Existing literature shows that eHealth methods can be of help in supporting health care workers in a crisis like COVID-19 (46). However, more research is needed about eHealth and delivering interventions online to understand which techniques work most efficiently (47). It should also be highlighted that populations at risk of infection, because of their age or economic class, may be at a disadvantage using eHealth services (48).

Counsellors had to quickly adapt to the new, online way of offering services while managing their personal life. They had to find the right focus for the 3 sessions, recognise the limits of the service and differentiate whether a client reports symptoms of a mental health disorder. Their response to crisis could be improved if the pre-service training for students in relevant fields included a stronger emphasis on crisis intervention and preparedness. Counsellors, and helping professionals broadly speaking, should have access to support with their own mental health to avoid burnout during crisis (49). Regular supervision could help them maintain their own mental health as well as reflect on how best the clients can be supported. Finally, future research on counselling services could investigate in depth how counsellors experience contextual changes in their working environment.

Referring cases beyond the competency of counselling proved to be a challenge due to the closure of specialist services. A solution to this could be designing socially distanced, emergency services for urgent cases needing mental health care.

## Conclusion

The COVID-19 pandemic brought about unique difficulties in the form of immense, rapid changes and little time to adjust. The COVID-19 counselling programme in Eötvös Loránd University, Hungary is an example of providing online mental health counselling support in the time of crisis. In this paper we summarised our observations regarding the experiences of clients, the features of the programme and experiences of counsellors. We saw in practise what a public health crisis can bring about in terms of its psychological and psychosocial impact. We learnt about the challenges and potentials of the online delivery of mental health care. Based on these experiences, we suggest that our task as mental health professionals is to help clients find meaning even in such unprecedented times. We can use the lessons learnt to further develop mental health services—for example by evaluating intervention effectiveness in crisis. Experience and knowledge sharing across practitioners should be encouraged, therefore improving how our field reacts to unexpected, high risk events, and crises. Our report about the COVID-19 counselling programme is contributing to this knowledge exchange and reflection.

## Data Availability Statement

The original contributions presented in the study are included in the article/supplementary material, further inquiries can be directed to the corresponding authors.

## Author Contributions

ZS and MK contributed to literature search, data analysis, and data interpretation. They wrote the manuscript and sent draughts to the rest of the team in iterations for comments and feedback. SB, PK, and AL contributed to data analysis and interpretation. OK provided overall feedback and comments on the earlier draughts and on the final manuscript. ZD contributed to the literature search and supervised the work and provided overall comments and feedback on the earlier draughts and on the final manuscript. All authors contributed to the article and approved the submitted version.

## Conflict of Interest

The authors declare that the research was conducted in the absence of any commercial or financial relationships that could be construed as a potential conflict of interest.
